# Decoding brain activities of literary metaphor comprehension: An event-related potential and EEG spectral analysis

**DOI:** 10.3389/fpsyg.2022.913521

**Published:** 2022-07-22

**Authors:** Lina Sun, Hongjun Chen, Chi Zhang, Fengyu Cong, Xueyan Li, Timo Hämäläinen

**Affiliations:** ^1^School of Foreign Languages, Dalian University of Technology, Dalian, China; ^2^Faculty of Information Technology, University of Jyväskylä, Jyväskylä, Finland; ^3^School of Biomedical Engineering, Faculty of Electronic Information and Electrical Engineering, Dalian University of Technology, Dalian, China

**Keywords:** literary metaphor, event-related potentials, N400, P200, neural oscillations

## Abstract

Novel metaphors in literary texts (hereinafter referred to as literary metaphors) seem to be more creative and open-ended in meaning than metaphors in non-literary texts (non-literary metaphors). However, some disagreement still exists on how literary metaphors differ from non-literary metaphors. Therefore, this study explored the neural mechanisms of literary metaphors extracted from modern Chinese poetry by using the methods of Event-Related Potentials (ERPs) and Event-Related Spectral Perturbations (ERSPs), as compared with non-literary conventional metaphors and literal expressions outside literary texts. Forty-eight subjects were recruited to make the semantic relatedness judgment after reading the prime-target pairs in three linguistic conditions. According to the ERPs results, the earliest differences were presented during the time window of P200 component (170–260 ms) in the frontal and central areas, with the amplitude of P200 for literary metaphors more positive than the other two conditions, reflecting the early allocation of attention and the early conscious experience of the experimental stimuli. Meanwhile, significant differences were presented during the time window of N400 effect (430–530 ms), with the waveform of literary metaphors more negative than others in the frontal and central topography of scalp distributions, suggesting more efforts in retrieving conceptual knowledge for literary metaphors. The ERSPs analysis revealed that the frequency bands of delta and theta were both involved in the cognitive process of literary metaphor comprehension, with delta band distributed in the frontal and central scalp and theta band in parietal and occipital electrodes. Increases in the two power bands during different time windows provided extra evidences that the processing of literary metaphors required more attention and effort than non-literary metaphors and literal expressions in the semantic related tasks, suggesting that the cognitive process of literary metaphors was distinguished by different EEG spectral patterns.

## Introduction

It is broadly agreed that metaphorical expressions in literary texts (hereinafter referred to as literary metaphors) are more novel, creative and richer in meaning compared with those outside literary texts (non-literary metaphors; Katz et al., 1988; Lakoff and Turner, 1989; Goatly, 1997; Semino and Steen, 2008). These researchers postulate that metaphorical expressions in literature, e.g., poetry, are used to extend our understanding of ordinary linguistic resources and bring fresh insights into human knowledge, but no consensus has been reached on how metaphor in literary texts is different from that in other communicative texts.

On the one hand, some previous studies (Nowottny, 1965; Leech, 1969; Tsur, 1992; Short, 1996) proposed the discontinuity between metaphor in and outside literature by addressing highly creative, original, and complex literary examples. They explored the uses of metaphor in specific genres, texts, or authors to demonstrate the effects of a particular linguistic choice in its original context. From this point of view, the distinctiveness of a particular use of literary metaphor is highlighted, while non-literary metaphors are considered as derivatives and less worthy of investigation (Semino and Steen, 2008). On the other hand, Lakoff and Johnson (1980) supported the view of continuity between literary and non-literary metaphors in light of Conceptual Metaphor Theory (CMT), which proposed that metaphor is not merely an adornment or entertaining device in human language but a linguistic and cognitive tool which reflects how an abstract and conceptual domain is cognitively structured. They conceived metaphor in everyday language as primary and metaphor in literature as the creative elaboration of ordinary, non-literary metaphor. CMT has resulted in the re-assessment of the role of metaphor in non-literary context and brought new insights into literary metaphor. Lakoff and Turner (1989) also posited that the metaphorical expressions created by poets were novel uses of conventional conceptual metaphors or everyday metaphorical expressions. They argued that these poets extended our way of thinking and expressions by applying creatively the same metaphorical tools to everyday language.

### Metaphor processing models

What is more, some psycholinguistic models were proposed to illuminate the neural mechanisms of metaphor comprehension. One of the frequently cited models is the Graded Salience Hypothesis (GSH; Giora, 1997), positing that it is the degree of salience instead of figurativeness that determines the precedence of access. Meanwhile, saliency is determined by the conventionality, frequency, familiarity and prototypicality of the words, phrases or sentences. The literal meaning of novel metaphors is accessed first because the figurative meaning is less salient than literal ones. In contrast, the figurative meaning of conventional metaphors, which is more salient than literal ones, is encoded before the literal meaning. Thus, as opposed to the traditional theories like Standard Pragmatic View (Grice, 1975; Searle, 1979) which attributed temporal priority to the literal meaning, the GSH conceived that the processing differences were not based on the distinction of literalness or figurativeness, but on the degree of salience (Giora, 1997, 2003; Giora and Fein, 1999). The Career of Metaphor model (Gentner and Bowdle, 2001; Bowdle and Gentner, 2005), offered a unified theoretical framework which illustrated whether metaphors were processed directly depended on the degree of conventionality and linguistic form. This model postulates that the comprehension process for conventional and novel metaphors are different. Novel metaphors are understood as comparisons. There is a shift from comparison with categorization in processing as metaphors become increasingly conventionalized (Gentner and Bowdle, 2001; Bowdle and Gentner, 2005; Arzouan et al., 2007; Lai et al., 2009).

### Event-related potentials and event-related spectral perturbations of metaphor processing

In recent decades, many researchers have explored the differences between the comprehension of the metaphorical and the literal expressions (Pynte et al., 1996; Arzouan et al., 2007; Mashal et al., 2007; De Grauwe et al., 2010; Diaz et al., 2011; Bambini et al., 2016). Focusing on literary metaphors, some research interests (Katz et al., 1988; Steen, 1994; Goatly, 1997; Reid and Katz, 2022) have been turned to the cognitive aspects of metaphor comprehension. For instance, Steen (1994) pointed out that literary metaphors differed from journalistic metaphors by measuring various dimensions in English and Dutch. Goatly (1997) found that English poetic metaphors were more novel than metaphorical expressions from other texts. In contrast, in Katz et al.’s (1988) study, two sets of literary and non-literary metaphors were analyzed on ten psychological dimensions, such as the degree of metaphoricity, comprehensibility, and the ease of interpretation, but no substantial differences were presented between the two types of metaphors. In spite of this, only a few studies (Arzouan et al., 2007; Rutter et al., 2012; Chen et al., 2016; Tang et al., 2017; Bambini et al., 2019) have attempted to explore literary metaphors through empirical methods.

Event-Related Brain Potentials, with prominently high temporal resolution, is often used to explore the time course of cognitive mechanisms in metaphor processing. The stimulus-locked ERP component of N400, a negative going component peaking around 400 ms, has been well-studied in recent years. It has been shown that the amplitude of N400 varies systematically with the processing of semantic information. The N400 component was also seen as an index of the ease or difficulty of retrieving stored conceptual knowledge related to a word (Kutas and Federmeier, 2000). Most metaphor studies claimed a higher amplitude of N400 for novel metaphors than conventional metaphors and literal expressions (Arzouan et al., 2007; Lai et al., 2009; De Grauwe et al., 2010; Obert et al., 2018). Meanwhile, many studies reported longer reaction time but lower accuracy in the semantic judgment tasks for novel metaphors than literal expressions (Arzouan et al., 2007; Coulson and Van Petten, 2007; Lai et al., 2009; De Grauwe et al., 2010; Bambini et al., 2019). Moreover, the graded N400 waveforms suggested that the difficulty of metaphor comprehension was associated with the complexity of conceptual mapping and information integration. Besides, some other studies also indicated that conventional metaphors and literal expressions elicited similar amplitudes of N400 (Iakimova et al., 2005; Arzouan et al., 2007) due to their high salience and familiarity. Although the N400 amplitude has been discussed a lot in figurative language studies, most results focused on the analysis of time domain and few studies (Ma et al., 2016; Li et al., 2022) have been reported on the domain of time-frequency through ERSPs.

The visual P200 component, peaking between 150 ms and 275 ms, is a positive-going potential, reflecting the early stages in lexical perception. P200 component is suggested to be correlated with contextual information, like sentence-level constraints or congruity related to target words (Coulson and Brang, 2010), the cognitive processes such as working memory (Lefebvre et al., 2005) and memory processing (Dunn et al., 1998). Some recent studies indicated that P200 component was associated with the early processes of high-level language comprehension, such as humor and irony (Regel et al., 2010; Li et al., 2022). For instance, Regel et al. (2010) showed that the P200 component was influenced by the contextual information of speakers’ characteristics in literal and ironic language processing. Some studies about metaphor comprehension (Landi and Perfetti, 2007; Schneider et al., 2014) proposed that P200 component was relevant to the ease of decision making in the meaningful judgment tasks. Others reported that P200 was closely associated with pictograph languages like Chinese characters instead of alphabetic languages (Xie et al., 2016). While the N400 component has been discussed extensively, the components preceding N400 have been rarely reported (Freunberger et al., 2007; Lai et al., 2019). Therefore, the component of P200 is well worth investigating in figurative language comprehension for its specific roles in the language perception network.

Based on traditional ERP studies, many studies have shown that neural oscillations perform an essential role in the modulation and generation of ERPs (Makeig et al., 2002; Gruber et al., 2004; Freunberger et al., 2007; Bastiaansen et al., 2008). For instance, Freunberger et al. (2007) evidenced that theta oscillation reflected the top-down regulating processes of memory and was partly involved in the modulation of P200 component. Bastiaansen et al. (2008) discovered that theta power was crucial in the retrieval of lexical semantic information. Compared with conventional ERP studies that focused on the dimension of time, event-related spectral perturbations (ERSPs) could offer a more comprehensive perspective on the analysis of time-frequency dimension, providing a better account of the electrophysiological responses evoked by visual stimuli. Although it is adopted in some language processing studies, the time-frequency dimensional analysis has been scarcely used in metaphorical studies.

### Hypotheses

The current study aims to explore the cognitive and neurophysiological underpinnings of literary metaphors by focusing on the variables on the time domain (ERPs components) and time-frequency domain (ERSPs) as electrophysiological responses to visual-evoked neural activations. Firstly, it is hypothesized that there would be significant differences among three language conditions in the early stage, such as the P200 component, of metaphor processing, because P200 was reported to be more significant in processing complex language materials (Zhao et al., 2011). Secondly, we expected a gradient of N400 amplitude in which literary metaphors would elicited the largest waveform, followed by non-literary metaphors and literal expressions. Unlike literal expressions and highly conventional metaphorical expressions, literary metaphors are novel and unfamiliar to the subjects, it would be more challenging to collect information for meaning integration (Tartter et al., 2002; Lai et al., 2009). Finally, we expected to seek more evidence on the relationship between literary metaphors and ERP responses, and the neural oscillations in different frequency bands.

## Materials and methods

### Subjects

Forty-eight undergraduates and postgraduates from Dalian University of Technology (Liaoning Province, China) were recruited as paid volunteers to participate in the experiment. It has been confirmed that none of the subjects had participated in any of the pretests in this study. All the subjects were right-handed native Chinese speakers, with normal or correct-to-normal vision and no history of neurological/psychiatric disorders or reading disabilities. Written consent form was obtained from all the participants. They were all informed of the instructions and procedures and were asked to minimize body movements, especially from the head, before the experiment. This experiment was approved by the Research Ethics Committee of Dalian University of Technology. Data from six participants were excluded in the statistic analysis due to low number of correct trials (*n* = 4, the ratio of correct trials is 0.34, 0.42, 0.5, and 0.44, respectively) and noisy EEG data (*n* = 2, the ratio of noisy trials is 0.58 and 0.56), leading to a final number of 42 participants (18 male, 24 female) for further analysis. Age ranged from 19 to 25 years old (*M* = 22.43).

### Materials

A total of 150 pairs of Chinese phrases with the structure of stimulus 1 (3–12 Chinese characters) to stimulus 2 (2–7 Chinese characters), in the form of prime to target, were selected for the ERP experiment. These stimuli consist of three categories: literary metaphors, non-literary metaphors, and literal expressions, with 50 in each group (see Table 1 for Sample Stimuli). Literary metaphors were natural language extracted from the original context of modern Chinese lyric poems. Comparatively, non-literary metaphors and literal expressions are generally from the news report in Chinese newspapers or magazines. Another group of 50 pairs of phrases which are unrelated in meaning are created as fillers.

**Table 1:** Sample stimuli in the ERP study.

Category	Prime	Target
Literary metaphor	一张金黄的心 A golden heart	九月 September
		冬季 Winter
Non-literary metaphor	树木的医生 The doctor of trees	啄木鸟 Woodpecker
		害虫 Pest
Literal expression	德国的首都 Capital of Germany	柏林 Berlin
		东京 Tokyo

This paradigm of prime-target pairs was adopted to examine whether metaphor-comprehension-related neural mechanisms were triggered or not (Sotillo et al., 2004). The prime (stimulus 1) consisted of metaphorical or literal expressions, followed by a target word (stimulus 2) consisting of words or phrases that could or could not be defined by the prime (Sotillo et al., 2004), e.g., “一张金黄的心 (A golden heart)”—“九月 (September)”/“冬季 (Winter).” (The prime-target pairs that are nonrelated in meaning function as fillers). The subjects were required to decide whether Stimulus 1 was accurately described by Stimulus 2 or not. On the one hand, the priming tasks were widely used in figurative language studies, such as allegorical sayings (Zhang et al., 2013) and humor processing (Li et al., 2022). On the other hand, this paradigm was connected with violations of semantic expectation (Kutas and Hillyard, 1980; Barber et al., 2002; Friederici, 2004; Khateb et al., 2010), attention and working memory (Harmony, 2013).

Prior to formal experiment, three pilot surveys were conducted to test the relatedness, figurativeness, and familiarity of the experimental materials. Firstly, 50 raters who did not participate in the formal experiment were enrolled to decide whether the two words or phrases of the prime-target pairs were related with each other in meaning (1 = unrelated, 2 = somewhat related, 3 = highly related). Based on the results, those stimuli rated by at least 90% of the participants as consistent in meaning were selected. After that, another 50 students were asked to judge the figurativeness of the selected stimuli on a 1–3 scale (1 = not figurative, 2 = somewhat figurative, 3 = highly figurative). Expressions with an average of <1.5 were chosen as literal expressions (50 pairs), whereas the expressions with an average of more than 2.5 were selected as literary metaphors and non-literary metaphors (100 pairs). On the final list of stimuli, those selected stimuli were rated by another group of 50 raters on a 1–3 scale (1 = unfamiliar, 2 = somewhat familiar, 3 = highly familiar) regarding their familiarity.

**Figure 1:**
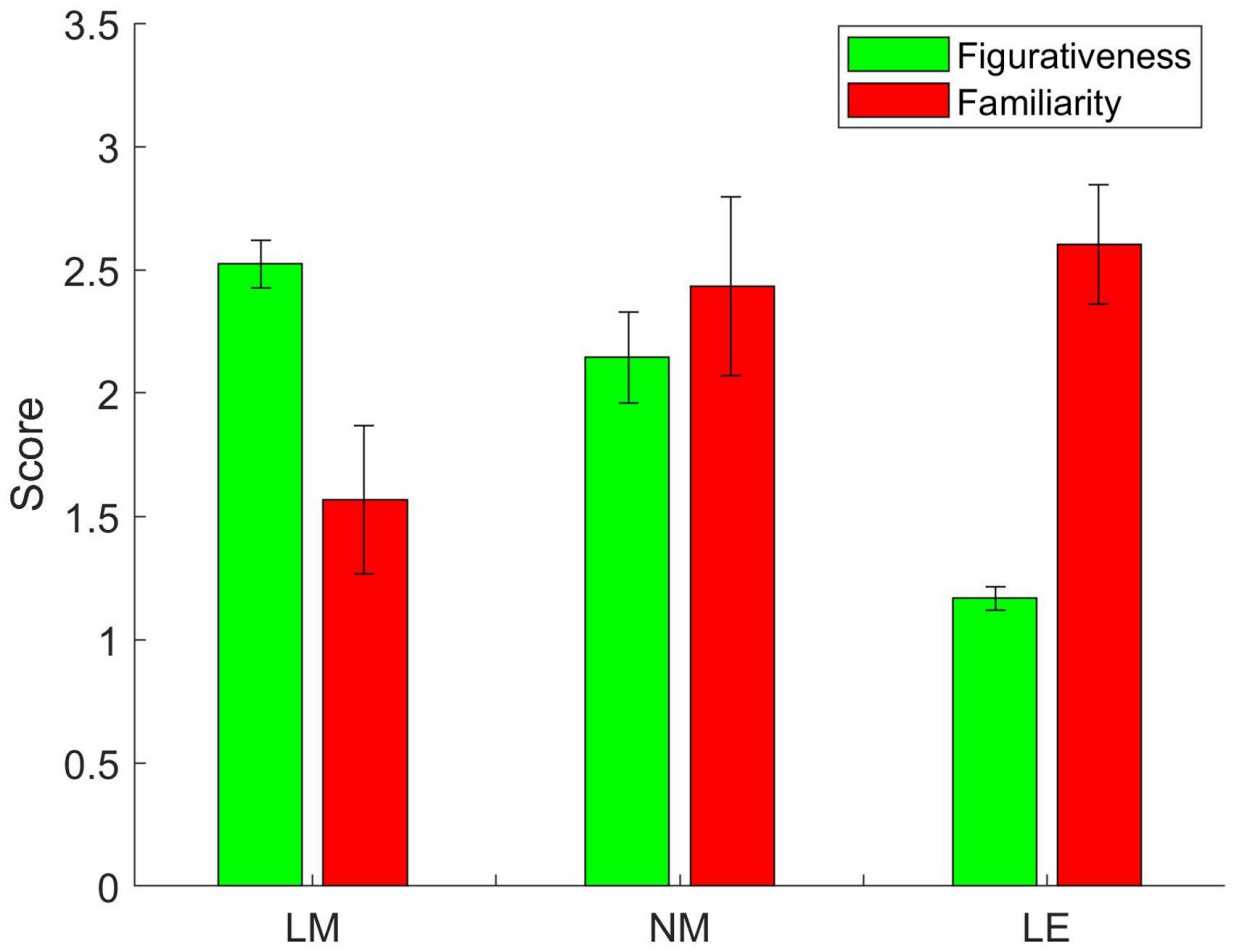
Figurativeness and familiarity of the experimental materials (LM, literary metaphors; NM, non-literary metaphors; LE, literal expressions).

The ANOVA (Analysis of variance) result shows that literary metaphors and non-literary metaphors are much more figurative than literal expressions, and there is a significant difference between the three groups, *F*(2, 147) = 1,594.04, *p* < 0.01. According to the result, both non-literary metaphors and literal expressions are more familiar than literary metaphors (Figure 1), and significant differences could be seen clearly between literary metaphors and non-literary metaphors, literary metaphors and literal expressions, *F*(2, 147) = 142.79, *p* < 0.01. Therefore, literary metaphors in lyric poems are more novel compared with those extracted from news report. In contrast, non-literary metaphors are highly conventional according to the result of pilot studies. Although literary metaphors are less familiar, they are still judged as meaningful by the raters, suggesting that literary metaphors are understandable instead of anomalous in meaning.

### Experimental procedure

During the whole ERP experiment, the subjects were instructed to sit in a dimly lit sound-attenuated chamber at ~80 cm from a 17-inch computer screen. All the stimuli were presented in white color on a black background. Following the experimental instructions, the subjects were required to read the prime-target pairs silently and judge whether the two words or phrases are semantically related to each other by pressing keys. All the experimental trials were displayed in a pseudo-randomized order to ensure that all the prime-target trials of the same type were not presented consecutively.

**Figure 2:**
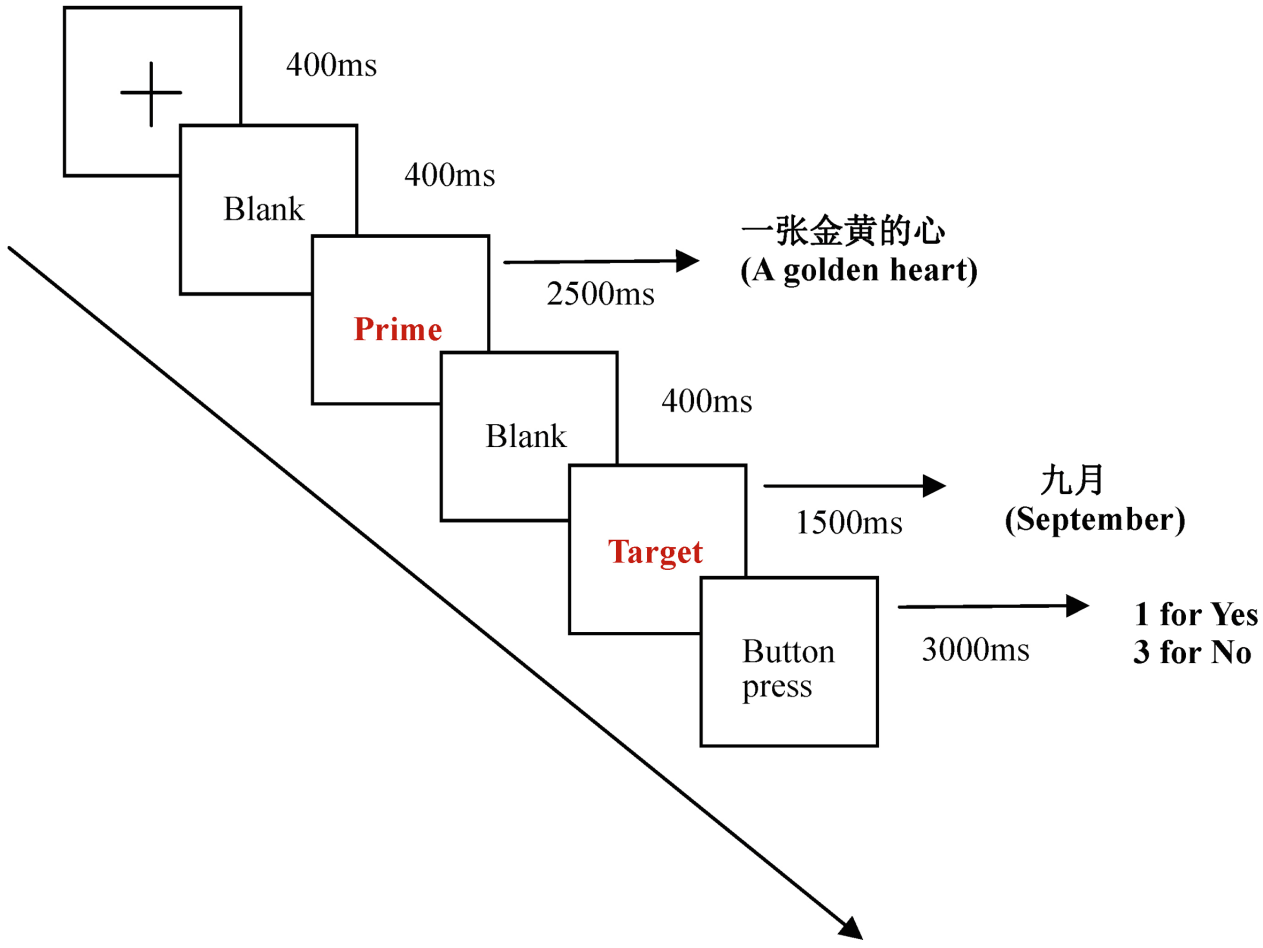
Experimental paradigm.

To get familiar with the task and procedure, the subjects were instructed with a brief practice of 15 trials of prime-target pairs, which were then not presented in the formal experiment. For each trial, the stimuli were presented in the following time sequence: fixation cross (400 ms), blank (400 ms), prime (2,500 ms), blank (400 ms), target (1,500 ms), and button press (3,000 ms). At the offset of the target word, a 3,000 ms reaction window would be presented. Upon seeing this screen, participants must judge whether these two words or phrases were semantically related (Yes/1, No/3). The inter-trial interval was 1,000 ms before a new trial starts. The overall sequence of events for a trial is illustrated in Figure 2. The formal experiment consisted of 50 trials for each category, with 50 fillers that are unrelated in meaning, leading to a number of 200 trials. The testing session was 30 min with two short breaks of 3 min. The accuracy and response time (RT) in the semantic judgment task were recorded. At last, the subjects with the accuracy rates of lower than 80% were excluded.

### EEG recordings and analysis

The EEGs were recorded with an electro cap of 64 Ag/AgCl electrodes according to the 10–20 System of electrode placement. An ANT Neuro EEG amplifier was used to record EEG signals sampled at a digitization rate of 500 Hz. The electrode impedance was kept below 5 kΩ, and the EEG was online referenced to the CPz channel.

In the offline analysis, EEG data were notch filtered at 50 Hz. Next, a digital high-pass filter of 0.5 Hz and a low-pass filter of 30 Hz were applied. After removing the direct current (DC) component, the data were re-referenced to the average of the mastoid references (M1, M2). The ERP epochs from 200 ms before to 1,300 ms after stimulus onset were extracted. Finally, by using the Icasso software (Himberg and Hyvarinen, 2003), independent artifact components (e.g., blinks, movements, etc.) were removed through visual inspection. Data of 6 subjects were excluded due to excessive artifacts.

### Event-related potentials

Event-related potentials were analyzed with MATLAB 2019b. First, the individual correct trials whose amplitudes were out of range (max >75 μv, baseline max >30 μv) were rejected, and then the baseline 200 ms before stimulus onset was subtracted from the waveforms. The equal number of trials for each subject under the conditions of LM, NM, and LE (LM = Literary Metaphors; NL = Non-literary Metaphors; LE = Literal Expressions) was adopted based on the minimum number of three condition trials. When the trial number exceeded the minimum number, the trials whose amplitudes were closer to the boundary of the range were removed. Next, trials were averaged across blocks for each subject. The total number of trials across all subjects for each condition was 1,185. The P200 and N400 amplitude and latency were quantified for further analysis. Based on the topographic activations, 15 electrodes (AF7, AF8, F5, F3, F1, Fz, F2, F4, F6, FC1, FCz, FC2, C1, Cz, and C2) were chosen for the N400 analysis. Three electrodes (AF7, AF3, and F5) were chosen for the P200 analysis. The time windows of 170–260 ms and 430–530 ms for the P200 and N400 components were selected. The N400 latency values were calculated as the time of maximum amplitude within the time window of the N400 component (Luck, 2014).

The significance level *p* < 0.05 was used, and all results were reported under the 2-tailed condition. One-way repeated-measures analysis of variance (ANOVA) with three language conditions (LM, NM, and LE) was used to test the hypothesis that LM initiates stronger effect on ERP components such as N400 and P200. Finally, the correlations between performance (accuracy, RTs, and omitted response) and ERP (the amplitude and latency of P200 and N400) were calculated using the Pearson Correlation Coefficient to investigate the association between the behavioral and electrophysiological measures in different language conditions.

### EEG spectra

The EEG spectral power was assessed by calculating the event-related spectral perturbation (ERSP) using the continuous wavelet transform (CWT; Guanghui et al., 2018). The complex Morlet wavelet was adopted for the CWT analysis, by which the time-dependent signals were evaluated at each sampling instant with a central frequency band of 1 Hz covering frequencies from 1 to 30 Hz, with a frequency step of 0.1 Hz. Additionally, we normalized the power spectra with the subtraction change from −500 to 0 ms baseline. For quantifying the oscillatory dynamics, we focused on separate time windows in the analysis of two frequency bands. According to the maximum power of the different frequency bands, statistical analysis was performed within the time window of 430–530 ms for the delta band (1–4 Hz) and within the time window of 170–260 ms for the theta band (4–8 Hz). In order to account for the effect of phase-locked (evoked response) activity in the induced oscillations, we also analyzed the induced activations by subtracting the averaged evoked response from each epoch prior to the wavelet analysis.

## Results

### Behavioral results

**Figure 3:**
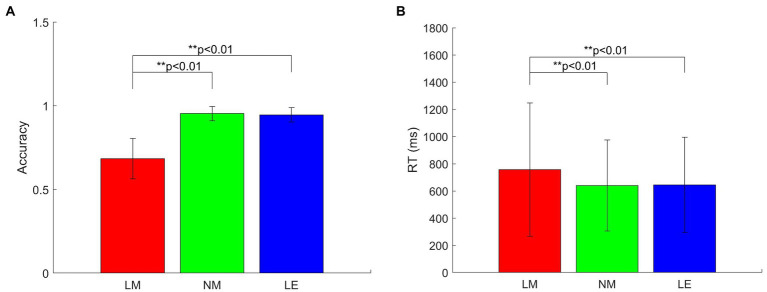
Behavioral performance. **(A)** Accuracy; **(B)** reaction time (RT). Averages and standard errors are plotted.

Figure 3 illustrates the behavioral performance (accuracy and RT) under different language conditions. Response accuracy was analyzed using one-way ANOVA. There was a significant effect of language conditions (*F*(2, 123) = 161.23, *p* < 0.01, $\eta_{p}^{2}=0.72$ ). Multiple comparison tests of Tukey’s honestly significant difference (Tukey’s HSD) procedure revealed that significant differences were reflected in the comparisons between LM and NM and between LM and LE (*p* < 0.01). Accuracy in the LM condition (mean = 0.6843, SD = 0.1210) was significantly lower than that of the NM condition (mean = 0.9529, SD = 0.0428) and that of the LE condition (mean = 0.9452, SD = 0.0424).

Response Times (RT) to the Probe in the correct trials were analyzed with a one-way ANOVA. There was a significant effect for language conditions (*F*(2, 4,287) = 39.48, *p* < 0.01, $\eta_{p}^{2}=0.02$ ). Multiple comparison test of Tukey’s HSD procedure revealed that significant differences were reflected in the comparisons between LM and NM and between LM and LE (*p* < 0.01). RT in the LM condition (mean = 756.33, SD = 489.92) was significantly longer than that of the NM condition (mean = 639.93, SD = 334.41) and that of the LE condition (mean = 644.06, SD = 349.47).

### Event-related potentials results

#### P200 component

**Figure 4:**
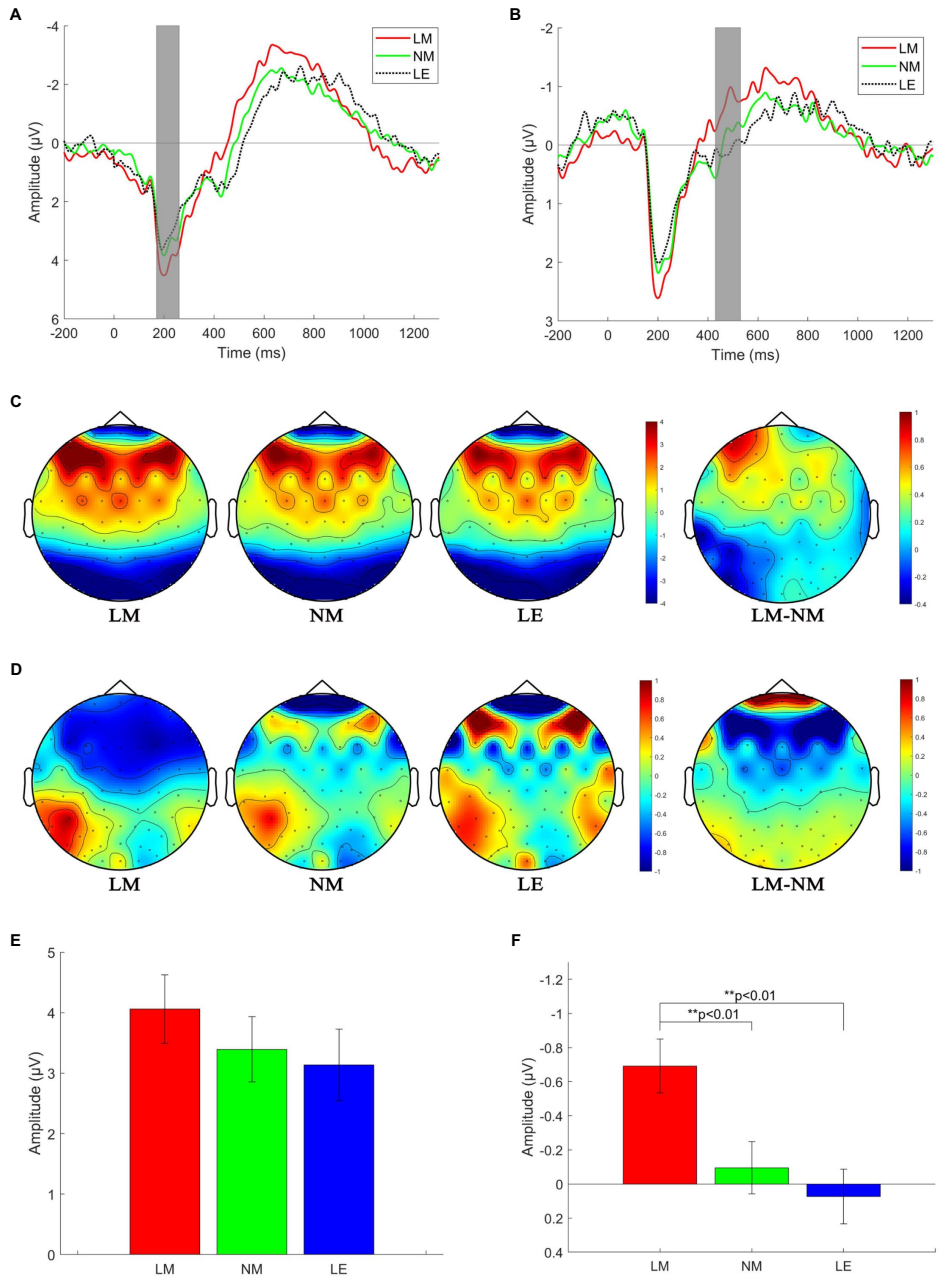
ERP responses to three stimulus conditions. **(A)** Grand average ERP of P200 channels. **(B)** Grand average ERP of N400 channels. **(C)** Topographies in P200 time window. **(D)** Topographies in N400 time window. **(E)** Mean values and standard error of the P200 amplitude in the three conditions. **(F)** Mean values and standard error of the N400 amplitude in the three conditions.

Figure 4A shows the averaged ERP amplitude waveforms with the time window of interest (P200 response at 170–260 ms after stimulus onset), depicted by the gray rectangle. Figure 4C shows the P200 topographies in three language conditions and the P200 topography difference between LM and NM. P200 activity is distributed in the frontal and central areas. The largest differences between LM and NM are mainly situated at the electrodes of AF7, AF3, and F5, where the P200 channels are selected. Figure 4E illustrates the mean values and standard error of the P200 amplitude in the three conditions. The one-way ANOVA reveals significant difference among three language conditions (*F*(2, 123) = 3.14, *p* < 0.05, $\eta_{p}^{2}=0.07$ ).

#### N400 components

Figure 4B shows the averaged ERP amplitude waveforms with the time windows of interest (N400 response at 430–530 ms after stimulus onset), depicted by the gray rectangles. Figure 4D shows the N400 topographies in the three language conditions and the N400 topography difference between LM and NM. N400 activity is distributed in large areas of the forebrain and mid-brain only in LM, which could hardly be detected in the condition of NM and LE. Figure 4F illustrates the mean values and standard error of the N400 amplitude.

The one-way ANOVA reveals a significant effect for language conditions (*F*(2, 123) = 6.44, *p* < 0.01, $\eta_{p}^{2}=0.09$ ). Based on the multiple comparison tests, we found the mean N400 amplitude of LM was larger than that of NM and LE. There was no statistically significant N400 response under the conditions of NM and LE.

### Event-related spectral perturbation results

**Figure 5:**
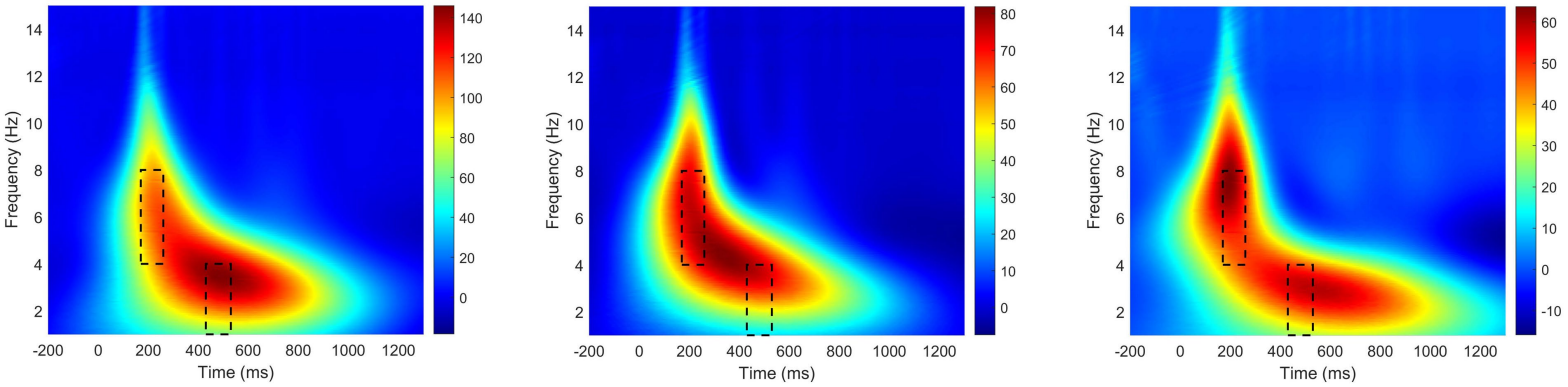
Time-frequency representations in the condition of LM, NM, and LE averaged over subjects.

**Figure 6:**
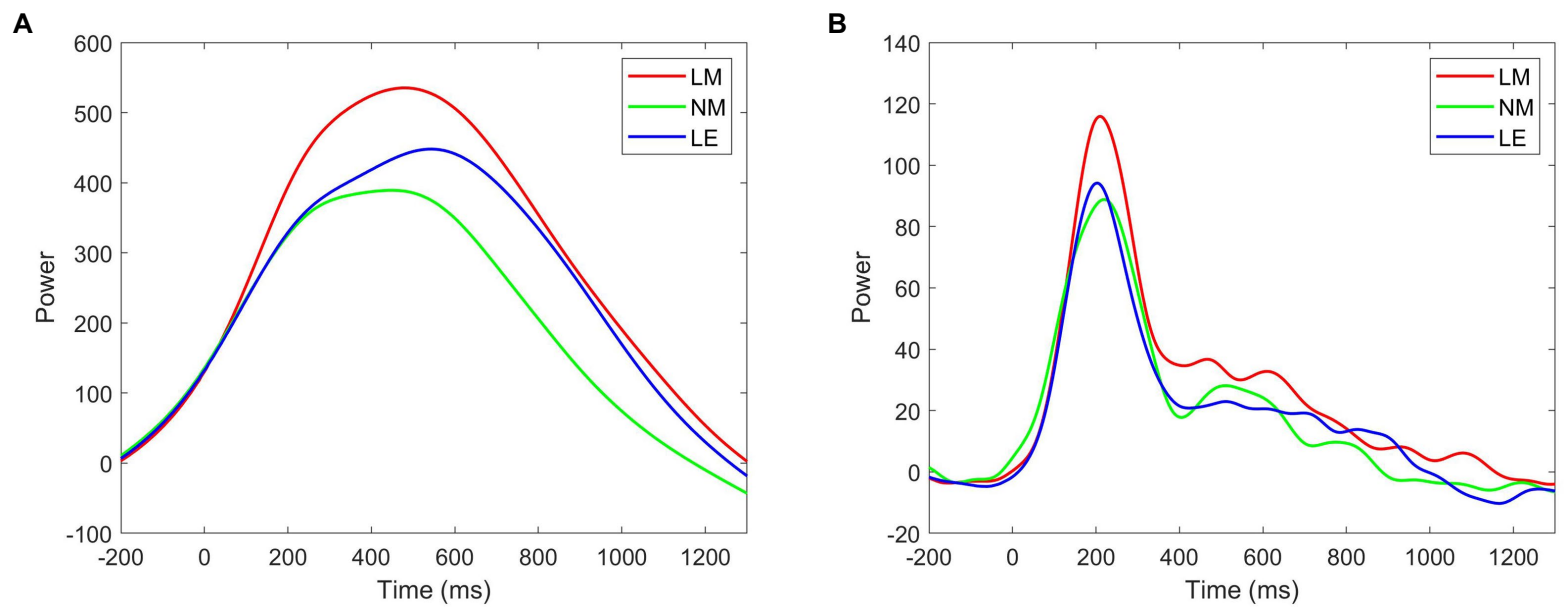
Temporal waveforms of power modulation. **(A)** delta; **(B)** theta.

**Figure 7:**
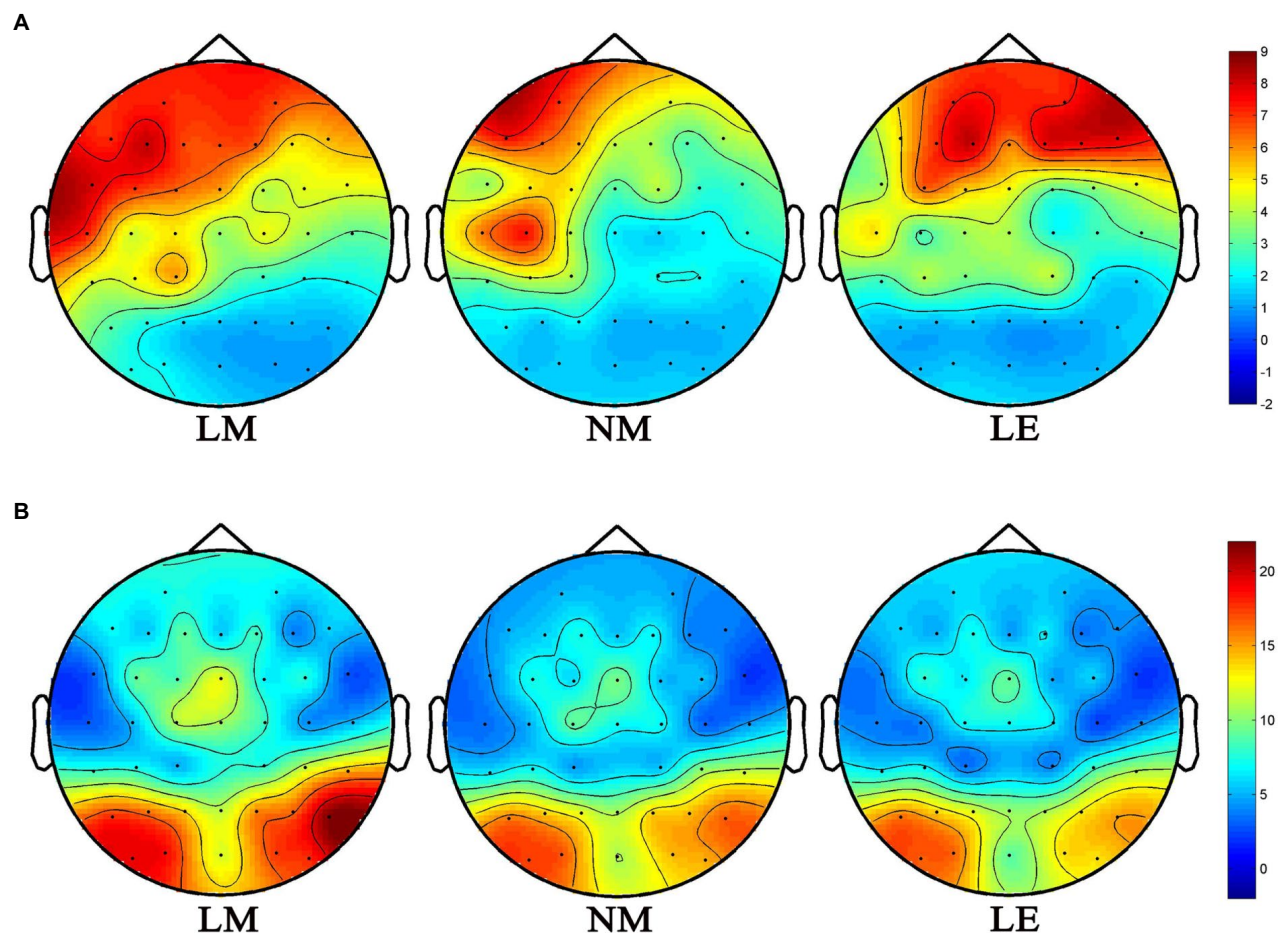
Topographies of power modulation. **(A)** delta; **(B)** theta.

Figure 5 illustrates the time-frequency representations (averaged over electrodes AF7, AF8, F5, F3, F1, Fz, F2, F4, F6, FC1, FCz, FC2, C1, Cz, and C2) in the three experimental conditions. A clear modulation of frequencies of 1–4 Hz is visible in the time window of 430–530 ms. Separable modulations of 4–8 Hz (in the time window of 170–260 ms) appear visually earlier than 1–4 Hz over the three conditions. The oscillations in other frequency bands are not activated with low power values. Therefore, only delta and theta are analyzed in this work. The corresponding frequency bands and time windows are indicated by the dotted-line boxes. Figures 6A, 7A show the power waveforms averaged across the electrodes (referred above) and topographic distribution corresponding to delta band (averaged over 1–4 Hz). The delta oscillations are mainly distributed in the frontal and central areas. Figures 6B, 7B show the power waveforms and topographic distribution of theta band (averaged over 4–8 Hz), with the strongest activations in the parietal and occipital electrodes.

With one-way ANOVA, for delta band power, we found a significant effect for language conditions (*F*(2, 123) = 7.51, *p* < 0.01, $\eta_{p}^{2}=0.11$ ) in the frontal and central areas. Based on the multiple comparison tests, we found that delta band power of LM was significantly higher than that of NM and LE. There was no statistically significant difference between the conditions of NM and LE.

For theta band, the significant effect for language conditions (*F*(2, 123) = 4.53, *p* < 0.05, $\eta_{p}^{2}=0.07$ ) is only found in the right parietal and occipital electrodes (P8 and PO8). Based on the multiple comparison tests, we found theta band power of LM was significantly higher than that of LE.

## Discussion

The main goal of the current study was to draw a clear picture on the cognitive process of novel metaphor comprehension by focusing on the study of literary metaphors from modern Chinese lyric poems. Specifically, this study was designed to evaluate the neural mechanisms of literary metaphors by analyzing the behavioral performances (accuracy and RT), the evoked responses (P200 component and N400 component), and spectral power (delta and theta bands).

### Behavioral data

Based on the results of behavioral performance, literary metaphors were proven to be significantly harder to comprehend correctly than non-literary metaphors and literal expressions (see Figure 3), in which the subjects took significantly longer time but achieved lower accuracy for the condition of literary metaphors. In the present study, the experimental stimuli in the group of literary metaphors were evidenced to be less familiar and harder to understand but still evaluated to be related in meaning, leading to the result that subjects spent more time making decisions. The research results were in line with those who reported longer response time and reduced accuracy for novel metaphors in relation to literal sentences (Coulson and Van Petten, 2007; Lai et al., 2009; De Grauwe et al., 2010). Comparatively, the subjects spent nearly the same amount of time in evaluating non-literary metaphors and literal expressions. Meanwhile, the response accuracy of these two language conditions were almost equal to each other. These results demonstrated that the subjects experienced similar comprehension process in approaching these two types of language materials. As a result, based on the behavioral performance, literary metaphors are revealed to be processed through significantly different ways compared with non-literary metaphors and literal expressions, while non-literary metaphors and literal expressions seem to experience similar language comprehension process. However, these results were not adequate to certify their differences. More information about the neurophysiological underpinnings of metaphor comprehension need to be considered.

### Event-related potential data

One of the more significant findings from this study is that the left-frontal P200 component is more positive for literary metaphors than non-literary metaphors and literal expressions. According to McDonough et al. (1992), the P200 component is not only considered to be an exogenous but also an endogenous component, which means that the P200 component may indicate the early sensory stages of item coding such as feature detection (Luck and Hillyard, 1994), selective attention (Hackley et al., 1990) and semantic processing (Federmeier and Kutas, 2002). Our research result about P200 component was compatible with Potts (2004), suggesting that the anterior P200 component was relative to task-relevant stimuli and was especially sensitive to the identification and judgment of experimental stimuli. Similarly, Kim et al. (2008) pointed out that difficult tasks could elicit significantly larger amplitude of P200 (stronger neural activities) than relatively easy tasks. Besides, some researchers (Naatanen and Näätänen, 1992) also reported that the P200 component reflected the early allocation of attention and the early conscious experience of the experimental stimuli. Therefore, the P200 effect was compatible with the assumption that more difficult priming tasks might evoke larger P200 amplitude (Kim et al., 2008; Zhao et al., 2011). In the current study, the stronger P200 effect distributed in the frontal region for literary metaphors might indicate the subjects were attempting to assess the relationship between prime and target words based on the task requirement during the early period of stimulus onset. Specifically, it could be reflected from P200 effect that literary metaphors were more challenging to process, requiring more attention and initial conscious awareness in language processing than non-literary metaphors and literal expressions.

However, this finding is inconsistent with the studies suggesting that P200 amplitude was stronger for the expected context than for the unexpected context (Wlotko and Federmeier, 2007). In other words, the stimuli with literal meaning should elicit a stronger P200 component than those with metaphorical sense. This inconsistency may be determined by the experimental paradigm in this study, the prime-target pairs, requiring subjects to make a decision about the relatedness of the two stimuli, which is quite different from the experimental stimuli in previous studies (Federmeier, 2007; Schneider et al., 2014; Li et al., 2022).

The second significant finding is that although there was a graded effect of the three language conditions on the N400 amplitude with literary metaphors eliciting the most negative waveform as reported by previous studies (Lai et al., 2009; Rutter et al., 2012), no significant differences were presented between non-literary metaphors and literal expressions. According to the behavior results, it is apparent from Figure 3 that the differences in accuracy and response time among the three language conditions were significant. The accuracy of literary metaphors is significantly lower, but the reaction time is much longer than the other two groups, in line with the ERP results that the N400 amplitude for literary metaphors is significantly larger than non-literary metaphors and literal expressions. This study suggested that it was more challenging to retrieve conceptual knowledge for literary metaphors than retrieving knowledge of non-literary metaphors or literal expressions in language comprehension. In other words, the construction of metaphorical mappings for literary metaphors was especially complex. The result of N400 responses was consistent with the features of different language materials, in which literary metaphors were always seen as more novel, unexpected and complicated in meaning, resulting in more efforts in establishing mappings between elements within distantly related domains (Arzouan et al., 2007; Goldstein et al., 2012). The interpretation of N400 component was in correspondence with the research finding of Arzouan et al. (2007); Coulson and Van Petten (2007); Lai et al. (2009); De Grauwe et al. (2010) and Bambini et al. (2019) who claimed longer reaction time and decline of accuracy regarding the meaningfulness judgment tasks for novel metaphors than literal expressions.

In terms of topographical distributions, it can be clearly seen that the N400 effect is distributed in the frontal and central area of the scalp for literary metaphors. This region is closely linked to language processing, indicating more efforts in lexical retrieval and semantic integration (Hald et al., 2006). The other two groups, in contrast, have presented few responses of N400. Furthermore, through the topographies of the N400 effect, more regions in the right part of prefrontal cortex were activated, which is reported to be related to figurative language processing (Seger et al., 2000). To this end, as previous neuroimaging studies evidenced (Beeman, 1993; Bottini et al., 1994), the right hemisphere may play a more significant role in connecting distantly related elements in metaphorical language comprehension. The right anterior regions of the N400 effect may signify the semantic integration of literary metaphorical relations. Thus, a further study will concentrate on the right hemisphere advantage in metaphorical language process through source localization algorithms.

According to the topographies during P200 and N400 time windows, significant differences could be observed among the three language conditions. Firstly, for the P200 effect, the topography difference between literary and non-literary metaphors is mainly distributed in the left frontal sites (see Figure 4C, LM-NM), which might play a critical role in the initial stage of meaning integration for literary metaphors. In contrast, no significant differences were found between non-literary metaphors and literal expressions, presenting nearly identical topographies for P200 effect. Secondly, for the N400 effect, the topography difference between literary and non-literary metaphors is mainly distributed in the frontal and central regions (see Figure 4D, LM-NM), which has been reported to be critical in many other studies of novel metaphor comprehension (Bambini et al., 2019; Li et al., 2022). Comparatively, no significant difference was presented between non-literary metaphors and literal expressions, indicating similar processing mechanisms in language comprehension (Iakimova et al., 2005; Arzouan et al., 2007). Meanwhile, our results were compatible with those that treat conventional metaphors as dead metaphors (Goldstein et al., 2012). To sum up, these results evidenced that literary metaphors are significantly different from non-literary metaphors and literal expressions in language comprehension process, while non-literary metaphors and literal expressions presented similar temporal dynamics during comprehension process. To this end, this study was in line with the research findings of Tang et al. (2017) and Bambini et al. (2019), illuminating the brain responses to literary metaphors were similar to those of novel metaphors instead of conventional metaphors.

### Event-related spectral perturbations data

The analysis of neural oscillations in EEG signal was proven to be a useful approach, since these data were almost lost in traditional time-locked ERP analysis by averaging single trials. In the present study, the result of ERSPs might help to clarify the neural mechanisms of metaphor comprehension to a greater extent.

Firstly, during the time window of 170–260 ms, literary metaphors were found to evoke significantly stronger increases in the frequency band of theta in comparison with non-literary metaphors and literal expressions in the parietal and occipital area (see Figures 6B, 7B), which is involved in visual cortex (Grill-Spector and Malach, 2004; Guzmán López et al., 2022), in the current study. Although literal expressions were slightly stronger in the frequency band of theta than non-literary metaphors, no significant differences were shown between these two groups (see Figure 6B). Theta increases have been reported to be related to the mental processes such as encoding and memory retrieval (Burgess and Gruzelier, 1997), working memory activation (Gevins et al., 1997; Krause et al., 2000; Deiber et al., 2007) and distribution of attention about target stimuli (Missonnier et al., 2006). Our research result is congruent with previous studies, on the one hand, the experimental design is relevant to visual tasks, as literary metaphors are less familiar and are more difficult to make a judgment, which always require more efforts in visual attention (Missonnier et al., 2006). On the other hand, theta power is associated with working memory process and the power increases with the difficulty of tasks (Weiss et al., 2000; Jensen and Tesche, 2002). Similarly, other researchers (Hagoort et al., 2004; Hald et al., 2006; Davidson and Indefrey, 2007) also claimed that changes of theta activity were connected to violations of semantic expectation, because more efforts were required and more attention were needed during semantic integration. In the present study, literary metaphor was shown to elicit stronger theta power than the other two language conditions, which is consistent with the research finding of Tesche and Karhu (2000), suggesting that theta power was sensitive to the encoding of novel stimuli.

Secondly, during the 430–530 ms time window, a significant effect can be clearly seen among three language conditions in the frequency band of delta, with delta power increases in literary metaphors being significantly larger than the other two conditions in the frontal and central areas of the topographical distributions (see Figures 6A, 7A). According to Fernández et al. (1993), the increase in delta oscillations may be associated with the increasing concentration in semantic evaluation tasks, suggesting that the subjects need to pay more attention to the comprehension of literary metaphors in relation to the other two language conditions. It was also reported that delta activity was consistent with the difficulty of experimental tasks, with more complex task elicit stronger delta power (Harmony et al., 1996). Similarly, as the topographical map shown, delta power mainly distributed in the frontal and central scalp, concurring with the ERP result during the N400 time window that the more difficult tasks (literary metaphors) evoked higher waveform of N400 amplitude. In this regard, our research result is in agreement with previous studies, indicating that delta power was significantly larger for literary metaphors during the time widow of N400, because literary metaphor, which was evidenced to take longer time for the subjects to comprehend, required more attention to deal with the complex activities.

In the current study, the changes in neural oscillations were reflected through different time-frequency topographic maps (see Figure 7), with theta band (4–8 Hz) allocated in the posterior electrodes and delta band (1–4 Hz) distributed in the central and frontal electrodes. Based on previous studies, both delta and theta oscillations functioned in the visual attention tasks (Knyazev, 2012; Keller et al., 2017). As a result, increases in the two power bands for literary metaphors during different time windows evidenced that literary metaphors required more attention and extra effort than non-literary metaphors and literal expressions in the semantic related tasks. In contrast, non-literary metaphors and literal expressions presented similar responses in neural oscillations.

This study tends to provide empirical evidence for the assumption that metaphors in literature are more novel and creative than metaphors outside literature (Semino and Steen, 2008), as readers pay more attention to metaphors in literary texts than to metaphors in non-literary texts (Glicksohn, 1994; Steen, 1994; Goodblatt and Glicksohn, 2002). The research results could be interpreted by the GSH. The salient meaning of non-literary metaphors is the figurative meaning, which could be immediately accessed, as there is no significant difference in lexical access of non-literary metaphors and literal expressions. In contrast, for literary metaphors, the literal meaning is the salient one, more contextual information should be reasonably inferred for the comprehension of the figurative meaning. Our results are also in line with the Career of Metaphor model, on the one hand, the conventionalized figurative meaning are processed through categorization instead of comparison, as there is an existing metaphorical category and no extra efforts were required in the comprehension process. On the other hand, novel metaphors, such as literary metaphors, are processed by establishing correspondences between partially isomorphic conceptual structures of the target and base. After encountering literary metaphors, the initial attempts at categorization failed due to lack of clearly defined category. Therefore, the comparison process begins after discovering that the literal meaning cannot be reasonably applied.

To sum up, this study is consistent with the perspective of continuity between metaphors in and outside literary texts. As Semino and Steen (2008) point out, the metaphorical uses of language need to consider both the uniqueness of specific uses in the language context and how particular uses interact with general conventional patterns, reflecting that the cognitive structures and process might be commonly shared by these two conditions. In other words, literary metaphors often have a conventional basis, and can be seen as extensions and elaborations of conventional metaphors (Semino and Steen, 2008).

### Limitations and outlook

Due to the difficulties in measuring electrophysiological responses to literary metaphors during naturalistic comprehension and the complexity of experimental design, the stimuli in this study are words and phrases extracted from the original context. Thus, a future study should be carried out by using more natural materials to examine their temporal and topographical characteristics. For instance, it was proposed that a continuous narrative could be used as experimental stimuli and the neural responses could be recorded along with the discourse. The metaphorical expressions would be time-locked for the analysis of temporal dynamics or frequency power (Bambini et al., 2019). It is a great challenge to move from word level to discourse level to comprehend the differences between literary and non-literary metaphors. Besides, individual differences in comprehending figurative language should be taken into consideration in future studies (Peskin, 2010; Abraham et al., 2021). Previous studies suggested that metaphor comprehension is a long-lasting process, greatly influenced by individual characteristics (Columbus et al., 2015). Accordingly, a further study could focus on the impact of individual differences on figurative language comprehension.

## Conclusion

The current study explored the neural mechanisms of literary metaphor comprehension by focusing on the temporal dynamics and neural oscillations of different types of language materials underlying the paradigm of prime-target pairs. Results presented a two-phase language processing procedure, with significantly stronger P200 followed by N400 for literary metaphor condition. Most ERP studies on metaphor comprehension reported N400 and P600 effects, with few studies discussing the role of P200 in metaphor processing. Meanwhile, the neural oscillations of three types of stimuli were in line with the responses of P200 and N400 waveforms. Increases for power bands of delta and theta were found for literary metaphors indicative of statistical differences between metaphors in and outside literature and literal expressions. As for topographical characteristics in ERP study, the frontal and central sites were critical in literary metaphor comprehension. While for the ERSPs results, the delta band and theta band during two different time windows were distributed in significantly different regions, reflecting different roles and functions of the two low-frequency power bands. To sum up, this study reveals a distinctive comprehension process for metaphors in and outside literary texts and literal expressions by uncovering the time courses and EEG spectral patterns. A future study will focus on the study of literary metaphors on discourse level and individual differences.

## Data availability statement

The raw data supporting the conclusions of this article will be made available by the authors, without undue reservation.

## Ethics statement

The studies involving human participants were reviewed and approved by the Research Ethics Committee of Dalian University of Technology. The patients/participants provided their written informed consent to participate in this study.

## Author contributions

HC and LS designed the experiment. CZ, LS, and XL analyzed and collected the data. CZ and LS conducted the statistics. LS and CZ wrote the manuscript. HC, FC, and TH revised the manuscript and provided guidance for all the conduction of work. All authors contributed to the article and approved the submitted version.

## Funding

This work was supported by “the Fundamental Research Funds for the Central Universities” under Grant DUT20RW401, Grant SIE18RZD2, and the scholarship from China Scholarship Council (No. 202207960001).

## Conflict of interest

The authors declare that the research was conducted in the absence of any commercial or financial relationships that could be construed as a potential conflict of interest.

## Publisher’s note

All claims expressed in this article are solely those of the authors and do not necessarily represent those of their affiliated organizations, or those of the publisher, the editors and the reviewers. Any product that may be evaluated in this article, or claim that may be made by its manufacturer, is not guaranteed or endorsed by the publisher.
