# Supplementary material for: Decoding brain activities of literary metaphor comprehension: An event-related potential and EEG spectral analysis
Source: Front Psychol. 2022 Jul 22;13:913521. doi: 10.3389/fpsyg.2022.913521 (PMC9356233; doi:10.3389/fpsyg.2022.913521)
Supplement: Supplementary file 1 [file Table_1.pdf]

Category	Stimulus 1	Stimulus 2
Literary metaphor<br>(*All the language materials in this study are only in Chinese.)	在黑暗中跳舞的心脏<br>a heart dancing in the dark	月亮/牛奶<br>moon/ milk
	两片抖动的小红帆<br>two shaking little red sails	嘴唇/牙齿<br>lips/ teeth
	一颗光芒四射的星辰<br>a radiant star	心脏/窗帘<br>heart/ curtain
	一串串熟透的眼泪<br>strings of ripe tears	湖泊/毛巾<br>lake/ towel
	一张金黄的心<br>a golden heart	九月/杯子<br>September/ cup
	河流幽幽的眼睛<br>the eyes of the river	灯/纸<br>lamp/ paper
	一面害羞的镜子<br>a shy mirror	水/脚<br>water/ foot
	拥抱太阳的手臂<br>arms hugging the sun	枝条/小船<br>branches/ boat
	一汪春池<br>a spring water	梦/伞<br>dream/ umbrella
	黄金的锚<br>gold anchor	新月/书本<br>a new moon/ book
Non-literary metaphor	和平的使者<br>messenger of peace	鸽子/麻雀<br>pigeon/ sparrow
	甜蜜的“健康杀手”<br>sweet "health killer"	糖类/白水<br>sugar/ water
	世界屋脊<br>roof of the world	青藏高原/月亮湾<br>Qinghai-Tibet Plateau/ the Moon Bay
	工业的血液<br>blood of industry	石油/森林<br>petroleum/ forest
	树木的医生<br>doctor of trees	啄木鸟/毛毛虫<br>woodpecker/ pest
	地球之肺<br>the lungs of the earth	雨林/煤矿<br>rainforest/ coalmine
	人际交往的金钥匙<br>the golden keys of interpersonal relationship	微笑/眼泪<br>smile/ teers
	人类的摇篮<br>cradle of humankind	地球/火星<br>Earth/ Mars
	知识的海洋<br>ocean of knowledge	大学/村庄<br>university/ village
	丰富的精神旅程<br>a rich spiritual journey	阅读/雨伞<br>reading/ umbrella

Literal expressions	飞机和火箭的结合体<br>a combination of airplane and Rocket	航天飞机/火车<br>space shuttle/ train
	世界著名喜剧大师<br>World Famous Comedy Master	卓别林/爱因斯坦<br>Chaplin/ Einstein
	主要的造血器官<br>major hematopoietic organ	骨髓/手臂<br>bone marrow/ arm
	航运的起点和终点<br>origin and destination of shipping	港口/汽车<br>harbor/ car
	没有配音配乐的电影<br>a film with no synchronized recorded sound	默片/谷物<br>silent film/ cereal
	农业生产的主体<br>people works under the umbrella of agriculture	农民/医生<br>farmer/ doctor
	新冠病毒的主要传播方式<br>the main transmission mode of the new coronavirus	飞沫传播/计算机<br>droplets in the air/ computer
	世界最大航天工程<br>the largest aerospace project in the world	国际空间站/珠穆朗玛峰<br>the International Space Station/ Mount Everest
	德国的首都<br>capital of Germany	柏林/女孩<br>Berlin/ girl
	儒家学派的创始人<br>the founder of Confucianism	孔子/学生<br>Confucius/ student
